# A systematic review and meta-analysis of the impact of triclosan exposure on human semen quality

**DOI:** 10.3389/ftox.2024.1469340

**Published:** 2024-10-17

**Authors:** Cecilia Adedeji Adegbola, Tunmise Maryanne Akhigbe, Adetomiwa Ezekiel Adeogun, Eva Tvrdá, Alica Pizent, Roland Eghoghosoa Akhigbe

**Affiliations:** ^1^ Department of Physiology, Ladoke Akintola University of Technology, Ogbomoso, Oyo State, Nigeria; ^2^ Reproductive Biology and Toxicology Research Laboratory, Oasis of Grace Hospital, Osogbo, Osun State, Nigeria; ^3^ Breeding and Genetics Unit, Department of Agronomy, Osun State University, Osogbo, Osun State, Nigeria; ^4^ Department of Physiology, Babcock University, Ilishan-Remo, Ogun State, Nigeria; ^5^ Institute of Biotechnology, Faculty of Biotechnology and Food Sciences, Slovak University of Agriculture in Nitra, Nitra, Slovakia; ^6^ Division of Occupational and Environmental Health, Institute for Medical Research and Occupational Health, Zagreb, Croatia

**Keywords:** endocrine disruptor, environmental toxicant, male infertility, oxidative stress, semen, sex hormones

## Abstract

**Introduction:**

Triclosan is an antibacterial and antifungal compound that is frequently found in personal care and consumer products, and its its impact on male reproductive health is a growing concern. Despite existing experimental studies demonstrating its potential threats to male fertility, reports on its effects on human semen quality remains limited and inconsistent. Therefore, this study presents a systematic review and meta-analysis assessing the relationship between triclosan exposure and semen quality.

**Methods:**

This study was registered with PROSPERO (CRD42024524192) and adhered to PRISMA guidelines.

**Results:**

The study analyzed 562 screened studies, out of which five articles including 1,312 male subjects were finally included in the study. The eligible studies were geographically diverse, with three from China, one from Belgium, and one from Poland. More so, the eligible studies were both case-control and cross-sectional. The meta-analysis revealed that triclosan exposure significantly reduced sperm concentration (Standard Mean Difference (SMD) −0.42 [95% CI: −0.75, −0.10], P = 0.01) and sperm total motility (SMD −1.30 [95% CI: −2.26, −0.34], P = 0.008). Mechanistic insights from animal and *in vitro* studies showed that oxidative stress may mediate the adverse effects of triclosan on semen quality.

**Discussion:**

This meta-analysis is the first comprehensive evaluation of the impact of triclosan on human semen quality, highlighting its potential to impair male fertility through reductions in sperm concentration and motility. However, the high heterogeneity among the included studies underscores the need for further high-quality research to establish more definitive conclusions regarding the effects of triclosan exposure on human reproductive health.

## 1 Introduction

**FIGURE 1:**
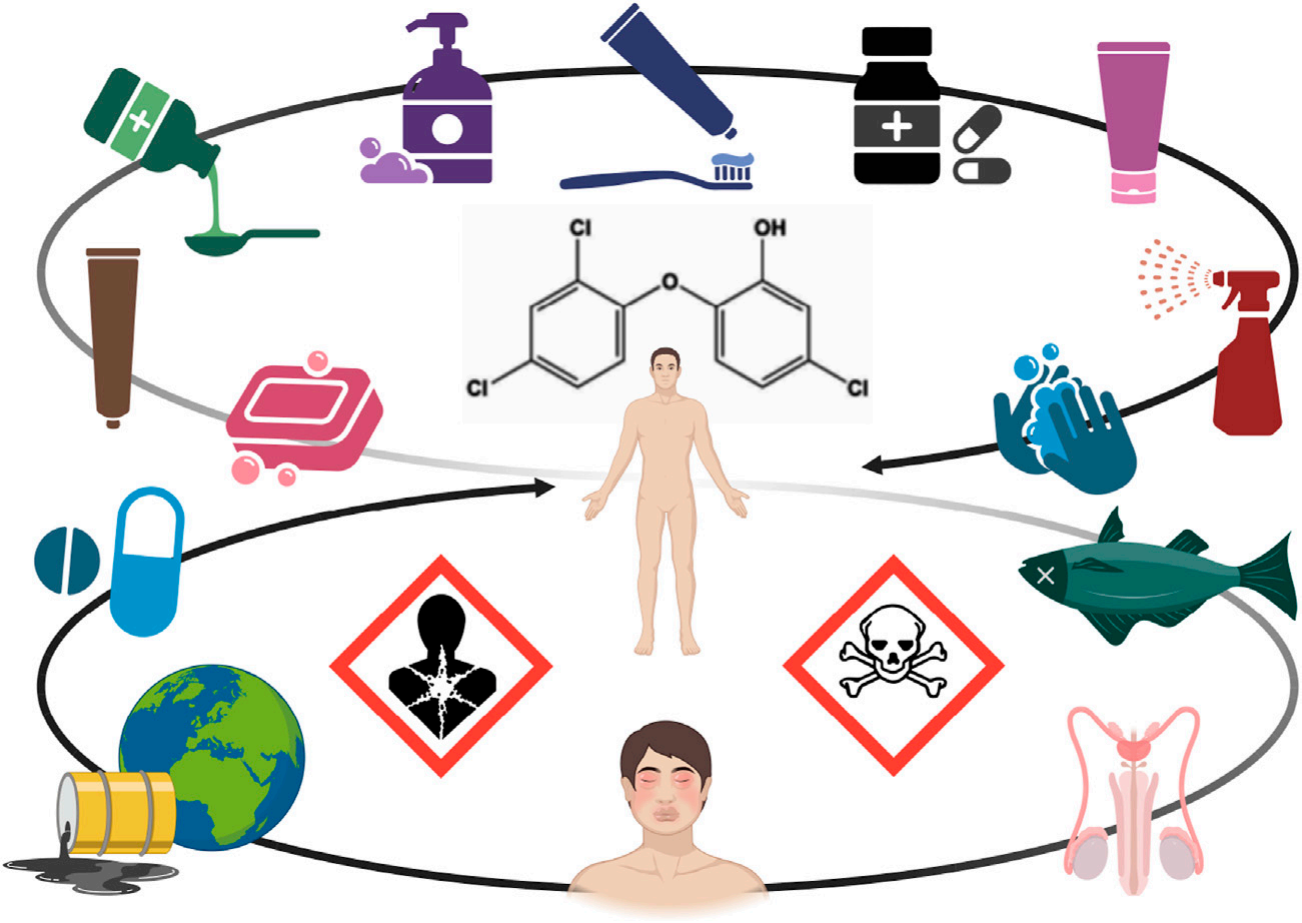
Sources and possible effects of triclosan. Triclosan may be found in common consumer products, including toothpastes, soaps, detergents, cleaning supplies, cosmetics, and medicaments. Acute or chronic exposure to triclosan may lead to acute or chronic toxicity, the occurrence of carcinogenic transformation products and resistance to antibiotics. Triclosan is considered as a persistent environmental pollutant capable of bioaccumulation, an allergen, and an endocrine disruptor.

Triclosan (5-chloro-2-[2,4-dichlorophenoxy]phenol) is a polychlorinated biphenolic antimicrobial and antifungal agent widely used in hygiene, personal care, and some consumer products. It is used to make soaps, mouthwashes, toothpaste, deodorants, cosmetics, dish detergents, foodstuff packaging materials, clothing, plastics, and children’s toys (Espitia et al., 2016; Weatherly and Gosse, 2017; Dar et al., 2022) (Figure 1). It is effective against gram-positive and gram-negative bacteria and therefore is used for disinfection of the hands, wounds, medical devices and equipment, and as a suture coating (Rašić et al., 2011; Otto-Lambertz et al., 2023). As a lipophilic molecule, triclosan is easily absorbed through skin and oral mucosa. Applied topically, triclosan entered the epidermis within the first hour of the experiment (Moss et al., 2000), and less than 10% of the administered dose from dermal spray and soap preparations was absorbed (Queckenberg et al., 2010). A single-dose oral intake of triclosan showed rapid absorption from the gastrointestinal tract (Sandborgh-Englund et al., 2006). The main route of triclosan excretion is urine with a median excretion half-life in humans of 11 h after oral intake (Moss et al., 2000; Sandborgh-Englund et al., 2006).

Due to its widespread use, it has been reported to contaminate water and food (Weatherly and Gosse, 2017; Dar et al., 2022; Sinicropi et al., 2022), contributing to its human exposure with possible attendant toxicity (Figure 1). It has been reported, mainly in experimental studies, that triclosan might disturb mitochondrial function, calcium signaling, and hormone homeostasis, as well as induce inflammation, lipid peroxidation, apoptosis, and oxidative stress (Weatherly and Gosse, 2017; Ruszkiewicz et al., 2017; Chen et al., 2023). Due to concerns for possible endocrine disrupting properties and potential to cause antibiotic/antimicrobial resistance and microbiome modification, the use of triclosan on food contact materials and in consumer antiseptic wash products is not allowed in the USA and the EU, whereas its use in cosmetics is under evaluation for endocrine disruption under EU legislation (Sinicropi et al., 2022; Halden et al., 2017). Furthermore, some information gaps about triclosan safety of repeated and multiple exposures to triclosan as well as its toxicity to animals and humans have been identified.

The greatest concern is related to triclosan’s potential endocrine disruption due to the close relationship between the functioning of the endocrine system and reproductive health and development. Its potential endocrine-disrupting effects related to reproductive health are mainly suggested by studies in aquatic and rodent models (Wang and Tian, 2015). Findings in aquatic species mainly suggested that triclosan has a weak estrogenic or antiestrogenic effect, whereas studies in non-human mammals indicated estrogenic or antiandrogenic potential of triclosan (Wang and Tian, 2015; Kumar et al., 2009). Using various cell lines, several authors found that triclosan may displace both estradiol and testosterone from their receptors and act as an agonist or antagonist, or no effect is observed (Wang and Tian, 2015; Kumar et al., 2008). It has been suggested that triclosan exposure may result in altered thyroid, estrogen, and androgen signaling pathways and decreased thyroid, luteinizing, and follicle-stimulating hormone, and testosterone, decreased semen production, impaired sperm morphology, and increased number of malformed sperms in treated animals (Kumar et al., 2009; Ena et al., 2018; Ha et al., 2018; Lan et al., 2015; Raj et al., 2021). However, some studies did not find any adverse effects of triclosan exposure (Zorrilla et al., 2009; Pernoncini et al., 2018).

Epidemiological studies on male reproductive health effects of triclosan are sparse and inconsistent. Several studies investigated the effects of triclosan exposure on couple fecundity (the ability of an individual to produce offspring) measured by time to pregnancy (TTP). Two studies of the same group of authors performed in couples from the USA found no association between triclosan concentration in urine (Smarr et al., 2017) or seminal plasma (Buck Louis et al., 2018) and TTP, whereas a study performed in couples from China found an inverse association between elevated male urinary triclosan and reduced fecundity expressed as a longer TTP in men with higher urinary triclosan (Zhu et al., 2022). Other authors evaluated fecundity or male fertility by measuring semen quality parameters and/or hormonal profiles in men and investigated the effects of triclosan on those parameters; these studies found no association between urinary triclosan and male infertility (Chen et al., 2013; Den Hond et al., 2015; Yuan et al., 2022) or found only a week impact of triclosan on semen quality parameters among men whose urinary triclosan concentration was in the first tertile (Zhu et al., 2016). Other studies found a significant association between triclosan exposure and abnormal sperm morphology (Jurewicz et al., 2018; Nassan et al., 2019), and increased sperm concentration and count (Smarr et al., 2018). Maternal exposure to triclosan during pregnancy was reported to increase neonatal cord blood testosterone and decreased estradiol (Wang et al., 2018), but there was no significant impact on serum testosterone in male US children and adolescents (Scinicariello and Buser, 2016) or adult males (Yan et al., 2020; Pollock et al., 2021), possibly due to very low urinary triclosan.

While some results suggest the adverse impact of triclosan exposure on male reproductive health, opposite results also exist. To allow more comprehensive insight into the potential adverse effects of triclosan exposure on male reproductive health, it is important to integrate available scientific evidence in systematic review methodology and to determine effect size across all of the studies by a meta-analysis (Johnson et al., 2016). Although several narrative reviews (Chiang et al., 2017; Green et al., 2021; Mínguez-Alarcón et al., 2023) and systematic reviews (Hipwell et al., 2019; Daza-Rodríguez et al., 2023) have been published recently, there is no meta-analysis on the impact of triclosan on semen quality at the time this report was documented. Therefore, this study provides the first comprehensive meta-analysis on the impact of triclosan exposure on human semen quality parameters. In addition, possible mechanisms underlying the potential toxic effects of triclosan on semen quality were systematically reviewed using available data from the literature to contribute to the understanding of how it may exert its toxicity in humans.

## 2 Materials and methods

This systematic review and its protocol were registered on PROSPERO (CRD42024524192). The work followed the Preferred Reporting Items for Systematic Reviews and Meta-analyses (PRISMA) standards (Shamseer et al., 2015). A thorough electronic search was undertaken on Google Scholar, Pubmed/Pubmed Central, and Scopus databases to discover relevant published papers from the databases until January 2024.

There was no restriction on language and study type. When conducting searches in the databases, MeSH terms were combined with other keywords and Boolean operators as (“triclosan” AND (“sperm” OR “spermatozoa” OR “sperm cell” OR “sperm concentration” OR “sperm count” OR “sperm viability” OR “sperm vitality” OR “sperm motility” OR “sperm morphology” OR “ejaculate volume” OR “semen volume”) AND (“follicle-stimulating hormone” OR “FSH” OR “luteinizing hormone” OR “LH” OR “testosterone” OR “male reproductive hormone” OR “male sex hormone”). Relevant full text articles and abstracts were retrieved. Also, the references cited in the relevant papers were manually retrieved.

### 2.1 Study selection and eligibility assessment

The eligibility criteria for included studies were determined strictly by the Population, Exposure, Comparator, Outcome, and Study Designs (PECO) framework (Morgan et al., 2018; Hamed M. A. et al., 2023). Accordingly, only studies in adult men of reproductive age (Population), assessing the impact of environmental exposure to triclosan, alone or in combination with a specified environmental toxicant (Exposure), in comparison with age-matched triclosan-unexposed adult men or the WHO standards for the outcomes (Comparator) on semen quality parameters (volume, sperm concentration, sperm count, total sperm motility, progressive sperm motility, and normal sperm morphology) and reproductive hormones (FSH, LH, and testosterone) (Outcomes) were eligible for inclusion in this review. These should be case-control, cohort, and cross-sectional studies (Study Design) that were able to adequately answer the question “What is the effect of triclosan exposure on human semen quality?”.

*In vitro* and animal studies, studies on prenatal triclosan exposure or exposure to environmental toxicants that do not include triclosan, reviews, meeting abstracts, studies lacking comparable variables, studies not reporting the actual values of the variables of interest in the form of mean and standard deviation or any other form from which the mean and standard deviation can be calculated, and studies documenting self-reported reproductive health outcomes were all excluded.

All authors participated in the search, but two (TMA and CAA) independently screened the titles and abstracts or the full text of the papers if an equivocal decision was made to retrieve relevant studies that meet the inclusion criteria. Disputes between the two reviewers were resolved by REA.

### 2.2 Data extraction and paper appraisal

The names of the authors, year of publication, country where the study was conducted, study design, the number of participants, their ages, triclosan concentration in the urine or seminal plasma, and the reported measured levels for the variables of interest were extracted from each paper. Furthermore, data on statistical analysis and adjustments, with results and conclusions was collected.

The quality of the evidence in studies that met the eligibility criteria was evaluated by the ErasmusAGE quality score for systematic reviews. The study design and size, methods of exposure measurement and outcome measurement, and analysis with adjustment were all evaluated. The following domains were scored: “study design (0 = cross-sectional study, 1 = longitudinal study, 2 = intervention study), study size (0 = < 50 participants, 1 = 50–150 participants, 2 = > 150 participants), method of measuring exposure (0 = not reported, 1 = moderate quality exposure, 2 = good quality exposure), method of measuring outcome (0 = no appropriate outcome reported, 1 = moderate outcome quality, 2 = adequate outcome quality), and analysis with adjustments (0 = no adjustments, 1 = controlled for one key confounder, 2 = additional adjustment for confounders” (Hamilton, 2008).

Three reviewers (TMA, CAA, and AEA) assessed the risk of bias (RoB), while the fourth reviewer (REA) handled any discrepancies that arose. The Office of Health Assessment and Translation (OHAT) tool was utilized to determine the RoB for each study (OHAT and NTP, 2015). Each domain was considered low risk of bias and scored one, or high risk of bias and did not receive any score. In addition, publication bias for studies included per variable of interest was determined by visually assessing the funnel plot (Hamed M. A. et al., 2023). The asymmetry of the funnel plot shows a potential publication bias.

The certainty of evidence was defined as confidence in the body of evidence and scored using the OHAT approach for systematic review and evidence integration in literature-based health assessment (OHAT and NTP, 2019). This adheres to the principles established by the Grading of Recommendations Assessment, Development, and Evaluation (GRADE) Working Group (GRADE Grading of Recommendations Assessment Development and Evaluation Working Group, 2014). Rooney et al. (2014) utilized four descriptive words; ‘high’, ‘moderate’, ‘low’, and ‘and ‘extremely low’ to describe the level of confidence.

### 2.3 Quantitative meta-analysis, and subgroup and sensitivity analysis

Review Manager (RevMan) software (version 5.4.1) was used to carry out a quantitative meta-analysis comparing triclosan-exposed persons to WHO reference standards that were used as controls (World Health Organization, 2010). Data from the eligible studies were pooled and the Standardized mean difference (SMD) and 95% confidence intervals for all reported variables were aggregated. The pooled analyses used the P-value and I-square statistic (I^2^) to estimate study heterogeneity, representing the proprotions of total variation across trials. If the P-value was less than 0.1 or the I2 -value was greater than 50%, a fixed-effects model was utilized; otherwise, a random-effects model.

To assess the sources of heterogeneity, we carried out subgroup analyses, excluding studies that did not have age-matched controls, hence only Den Hond et al. (Den Hond et al., 2015) and Guo et al. (Guo et al., 2023) were included. Furthermore, sensitivity analyses were performed by excluding the studies with the largest weight for each variable studied.

### 2.4 Mechanisms of triclosan reproductive toxicity

In addition, to explore the potential associated mechanisms linking triclosan and semen quality, a detailed review of *in vitro* studies in humans and animal studies was done. This is because of a lack of mechanistic studies in humans reporting the effect of triclosan on semen quality.

## 3 Results

### 3.1 Study characteristics

**FIGURE 2:**
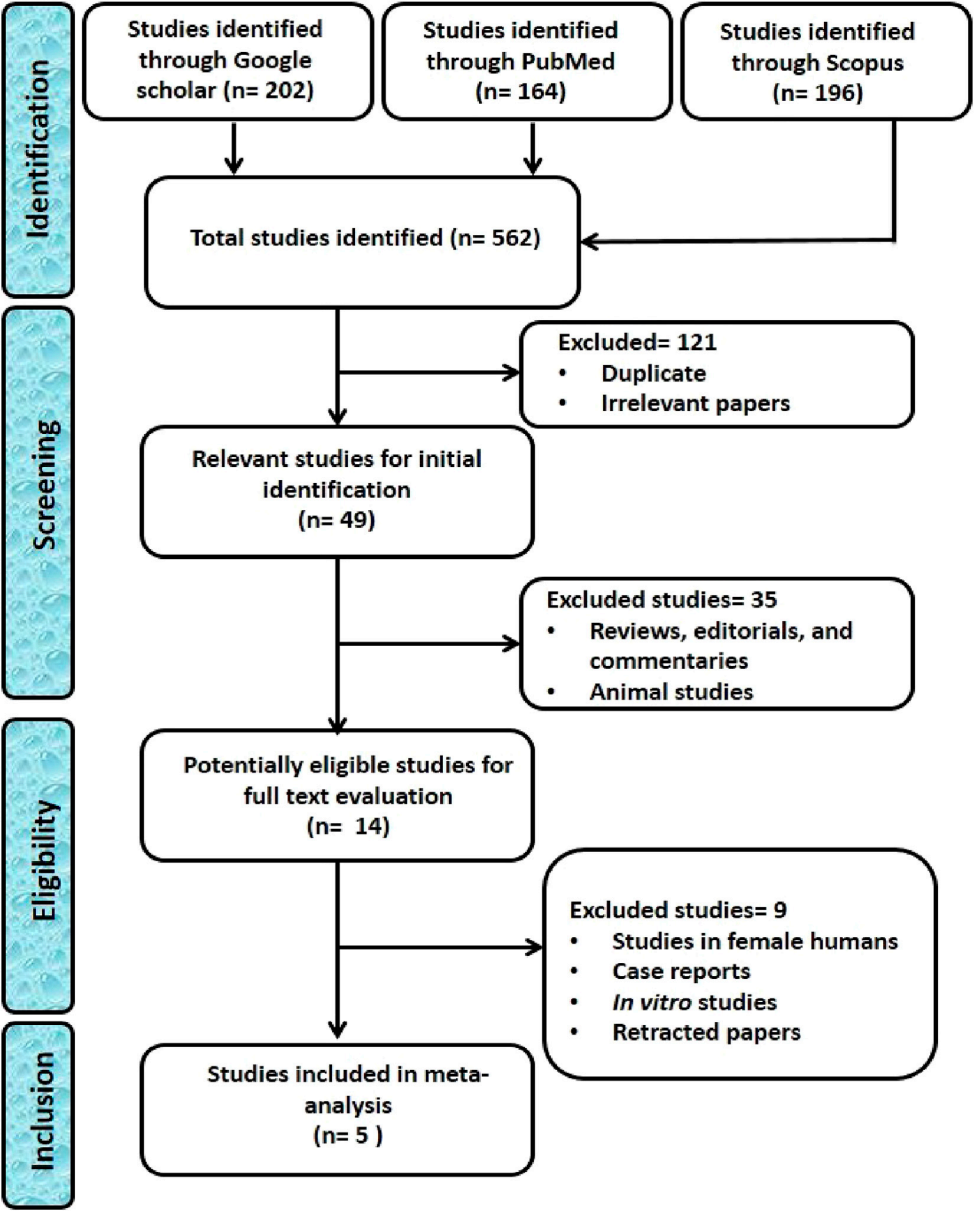
PRISMA flow chart for the selection of eligible studies on the impact of triclosan exposure on human semen quality.

Out of the 562 studies collected, only 5 were eligible for inclusion in the study (Figure 2). The eligible studies were from Belgium (1), China (3), and Poland (1). Two of these (Den Hond et al., 2015; Guo et al., 2023) were case-controlled, while others (Yuan et al., 2022; Zhu et al., 2016; Jurewicz et al., 2018) were cross-sectional studies, and a total of 1,312 male subjects were included in the meta-analysis.

Total triclosan concentration was determined in a spot urine sample in the four studies (Den Hond et al., 2015; Yuan et al., 2022; Zhu et al., 2016; Jurewicz et al., 2018), using either gas chromatography (GC-MS) (Den Hond et al., 2015; Jurewicz et al., 2018) or ultrahigh performance liquid chromatography coupled with tandem mass spectrometry (UPLC-MS/MS) (Yuan et al., 2022; Zhu et al., 2016). The results were then adjusted for urinary creatinine concentration. Only one study (Guo et al., 2023) assessed triclosan exposure by measuring triclosan in seminal plasma samples using the UPLC-MS/MS method. Unadjusted levels of triclosan in urine and seminal plasma are shown in Table 1.

**TABLE 1:** Characteristics of the eligible studies on the impact of triclosan exposure on human semen quality.

References	Study design	Country	Studied population	Age	Triclosan concentration Mean ± SD, Median (P25; P75) or GM (P25; P75) (Exposure sample)	Outcomes
Den Hond et al. (2015)	Case-control	Belgium	Control = 40 Case = 80	<50 years	Control: 2.8 (0.5; 5.5) µg/L Case: 2.6 (0.5; 15.6) µg/L (urine)	Sperm concentration, motility, and morphology
Guo et al. (2023)	Case-control	China	Control = 100 Case = 100	20–45 years	Control: 2.6 ± 11.9 μg/L Case: 4.0 ± 9.5 μg/L (seminal plasma)	Sperm count, concentration, total motility, and progressive motility
Jurewicz et al. (2018)	Cross sectional	Poland	Cohort = 315	<45 years	2.83 (0.84; 21.49) µg/L (urine)	Sperm concentration, motility and sperm with abnormal morphology
Yuan et al. (2022)	Cross sectional	China	Cohort = 406	18–60 years	1.7 (<0.5; 5.1) µg/L (urine)	Semen volume, sperm concentration, count, total motility, and progressive motility
Zhu et al. (2016)	Cross sectional	China	Cohort = 471	?	1.12 (0.50; 3.38) µg/L (urine)	Semen volume, sperm concentration, count, motility and normal sperm morphology

Case - male partners of couples that unsuccessfully tried to obtain pregnancy during ≥12 months and/or with semen quality parameters below the reference values.

SD, standard deviation; P25–25^th^ percentile; P75–75^th^ percentile.

Semen quality parameters were determined following the World Health Organization protocols (World Health Organization, 2010).

### 3.2 Assessment of the quality of evidence, RoB, and certainty of evidence

Based on the ErasmusAGE quality score for systematic reviews, two of the eligible studies (Yuan et al., 2022; Zhu et al., 2016) scored 80% (8/10), two (Den Hond et al., 2015; Jurewicz et al., 2018) scored 70% (7/10), and one study (Guo et al., 2023) scored 60% (6/10) (Table 2). This suggests that the quality of evidence of the eligible studies was good enough.

**TABLE 2:** Quality of evidence of the eligible studies on the impact of triclosan exposure on human semen quality.

References	Study design	Study size	Method of measuring exposure	Method of measuring outcome	Analysis with adjustment	Total
Den Hond et al. (2015)	1	1	1	2	2	7/10
Guo et al. (2023)	0	2	1	2	1	6/10
Jurewicz et al. (2018)	0	2	1	2	2	7/10
Yuan et al. (2022)	0	2	2	2	2	8/10
Zhu et al. (2016)	0	2	2	2	2	8/10

Using the OHAT RoB tool, all eligible studies had 5/9, except one (Guo et al., 2023) that had 4/9 (Table 3). This reveals that the included studies were not of high RoB. In addition, the studies included for semen volume, total motility, and normal sperm morphology showed a symmetry in distribution on the funnel’s plot suggesting that there was no publication bias. However, the studies included for sperm count, concentration, and progressive motility were asymmetric in distribution, suggesting publication bias (Supplementary Figure S1).

**TABLE 3:** Risk of bias of the eligible studies on the impact of triclosan exposure on human semen quality.

References	Representa-tiveness of exposed cohort	Selection of non-exposed cohort	Ascertainment of exposure	Outcome not present at start of study	Control for basic factors	Control for other factors	Assessment of outcome	Sufficient follow-up for outcome	Adequacy of follow-up	Total
Den Hond et al. (2015)	1	-	-	1	1	1	1	-	-	5/9
Guo et al. (2023)	1	-	-	1	1	-	1	-	-	4/9
Jurewicz et al. (2018)	1	-	-	1	1	1	1	-	-	5/9
Yuan et al. (2022)	1	-	-	1	1	1	1	-	-	5/9
Zhu et al. (2016)	1	-	-	1	1	1	1	-	-	5/9

Following the GRADE guideline for confidence in the body of evidence, two of the included studies (Den Hond et al., 2015; Guo et al., 2023) showed high confidence levels, while others (Yuan et al., 2022; Zhu et al., 2016; Jurewicz et al., 2018) showed moderate confidence levels (Table 4). It is worth noting that those with high confidence levels were the controlled studies, while those with moderate confidence levels were the cohort studies.

**TABLE 4:** Certainty of evidence of the eligible studies.

References	Initial assessment	Decreasing factors	Increasing factors	Final assessment
Den Hond et al. (2015)	Moderate	–	+	High
Guo et al. (2023)	Moderate	–	+	High
Jurewicz et al. (2018)	Low	–	+	Moderate
Yuan et al. (2022)	Low	–	+	Moderate
Zhu et al. (2016)	Low	–	+	Moderate

–: No influencing factor; +: presence of an influencing factor.

### 3.3 Assessment of the impact of triclosan on human semen quality

#### 3.3.1 Semen volume

**FIGURE 3:**
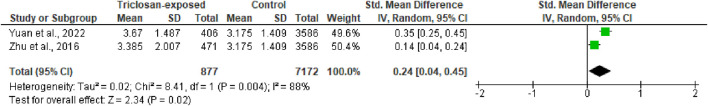
Forest plot showing the effect of triclosan exposure on ejaculate volume (mL).

Only two studies were eligible for inclusion in the assessment of the effect of triclosan on semen volume. Interestingly, triclosan significantly increased ejaculate volume (SMD 0.24 [95% CI: 0.04, 0.45] P = 0.02), with significant inter-study heterogeneity (I^2^ = 88%; X^2^ *p* = 0.004) (Figure 3).

#### 3.3.2 Sperm count

**FIGURE 4:**
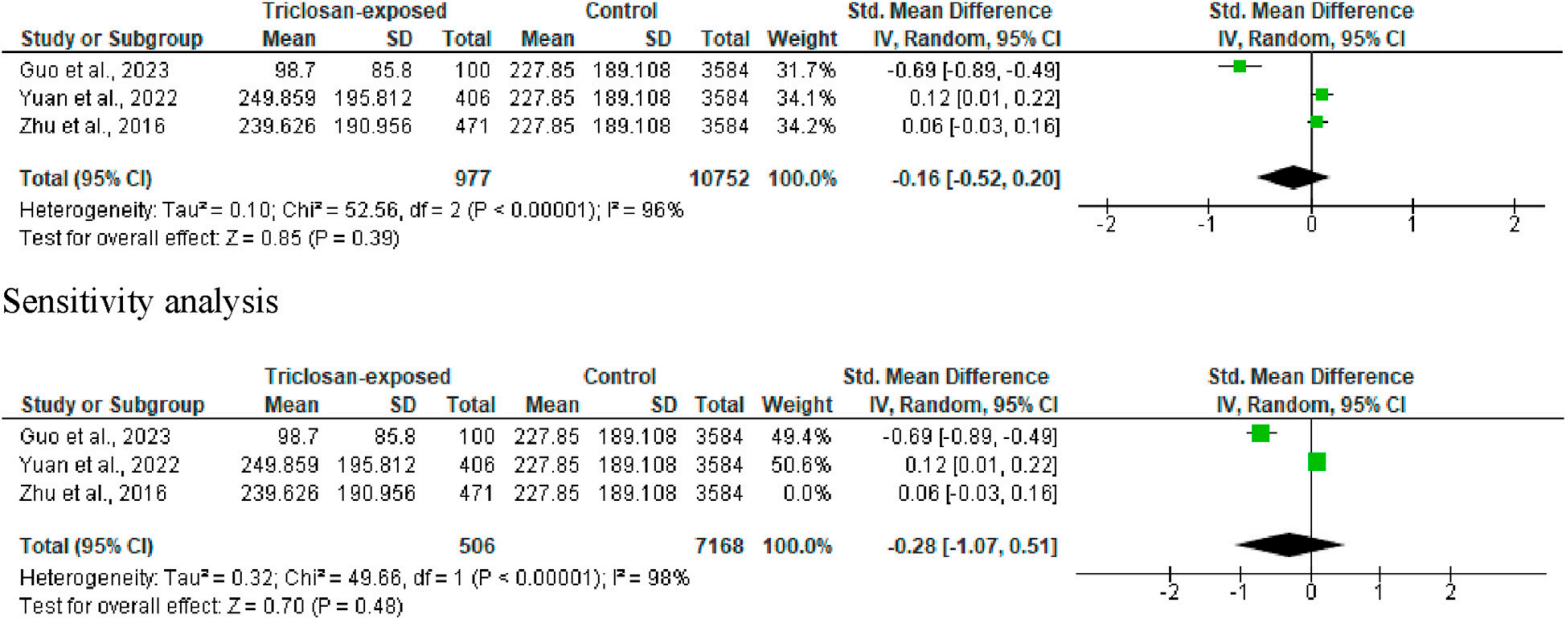
Forest plot showing the effect of triclosan exposure on sperm count (million/ejaculate).

Three studies were included in the analysis of sperm count. It was observed that triclosan did not significantly alter sperm count (SMD -0.16 [95% CI: 0.52, 0.20] *p* = 0.39). Also, there was a significant inter-study heterogeneity (I^2^ = 96%; X^2^ *p* < 0.00001). After sensitivity analysis, triclosan did not significantly alter sperm count (SMD -0.28 [95% CI: 1.07, 0.51] *p* = 0.48), and the significant inter-study heterogeneity persisted (I^2^ = 98%; X^2^ *p* < 0.00001) (Figure 4).

#### 3.3.3 Sperm concentration

**FIGURE 5:**
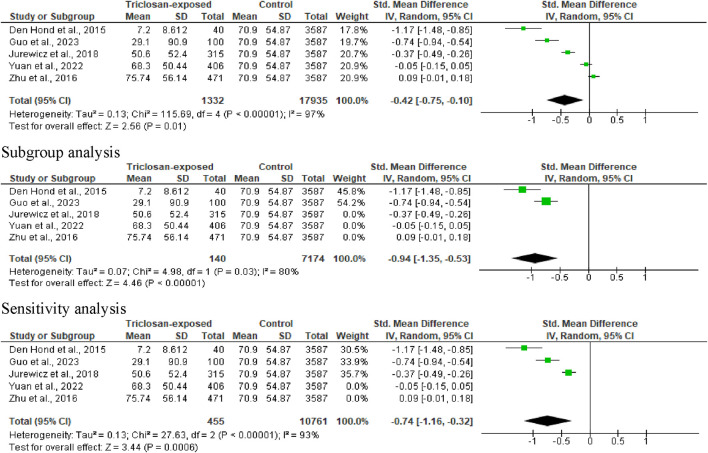
Forest plot showing the effect of triclosan exposure on sperm concentration (million/mL).

All eligible studies reported data on sperm concentration. Triclosan significantly reduced sperm concentration (SMD -0.42 [95% CI: 0.75, −0.10] *p* = 0.01), with a significant inter-study heterogeneity (I^2^ = 97%; X^2^ *p* < 0.00001). The observed considerable difference persisted even after subgroup analysis (SMD -0.94 [95% CI: 1.35, −0.53] *p* < 0.00001); (I^2^ = 80%; X^2^ *p* = 0.03) and sensitivity analysis (SMD -0.74 [95% CI: 1.16, −0.32] *p* = 0.0006); (I^2^ = 93%; X^2^ *p* < 0.00001) (Figure 5).

#### 3.3.4 Total sperm motility

**FIGURE 6:**
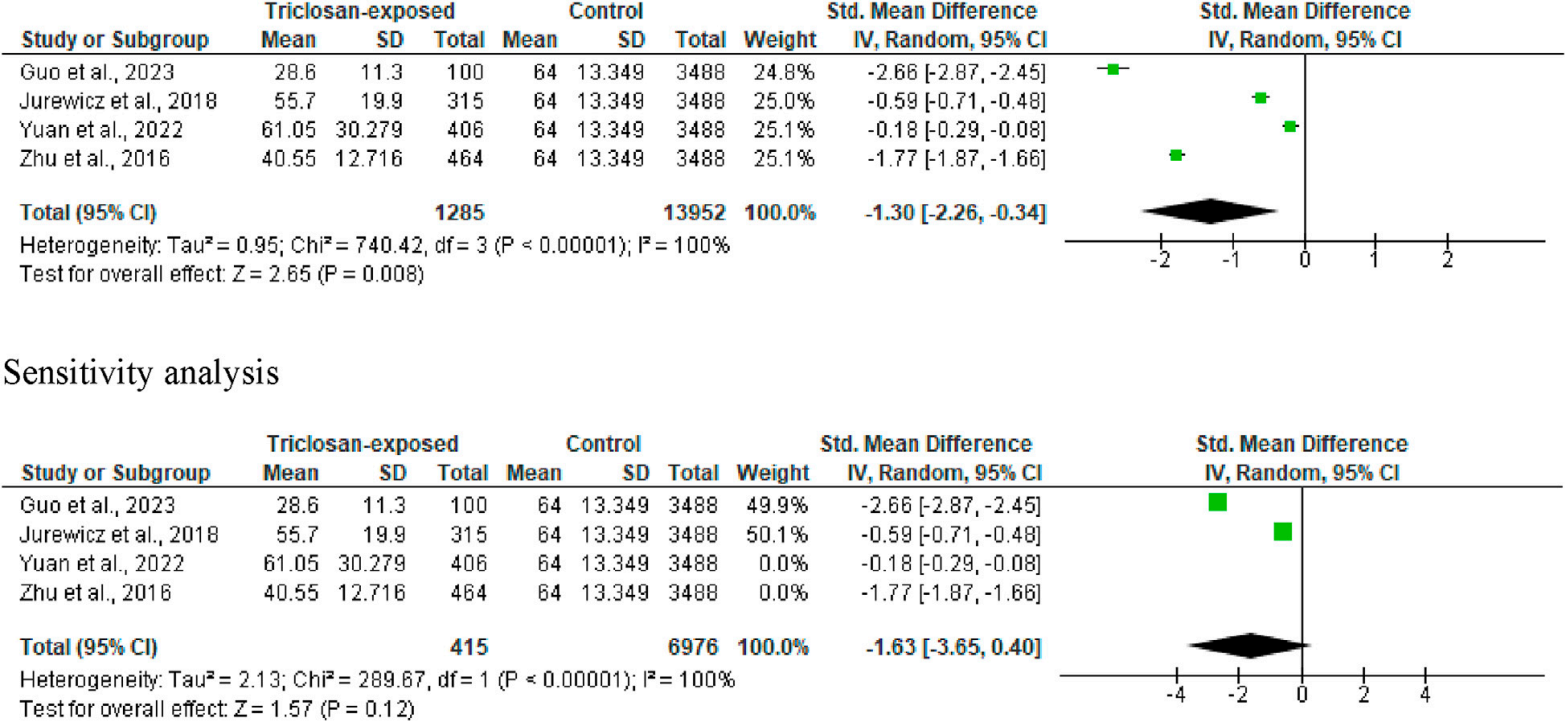
Forest plot showing the effect of triclosan exposure on total sperm motility (%).

Four out of the eligible studies were included in the analysis of total sperm motility. Triclosan significantly reduced total sperm motility (SMD -1.30 [95% CI: 2.26, −0.34] *p* = 0.008). More so, there was a significant inter-study heterogeneity (I^2^ = 100%; X^2^ *p* < 0.00001). However, the observed significant difference faded out with sensitivity analysis (SMD -1.63 [95% CI: 3.65, 0.40] *p* = 0.12), while the significant inter-study heterogeneity persisted (I^2^ = 100%; X^2^ *p* < 0.00001) (Figure 6).

#### 3.3.5 Progressive sperm motility

**FIGURE 7:**
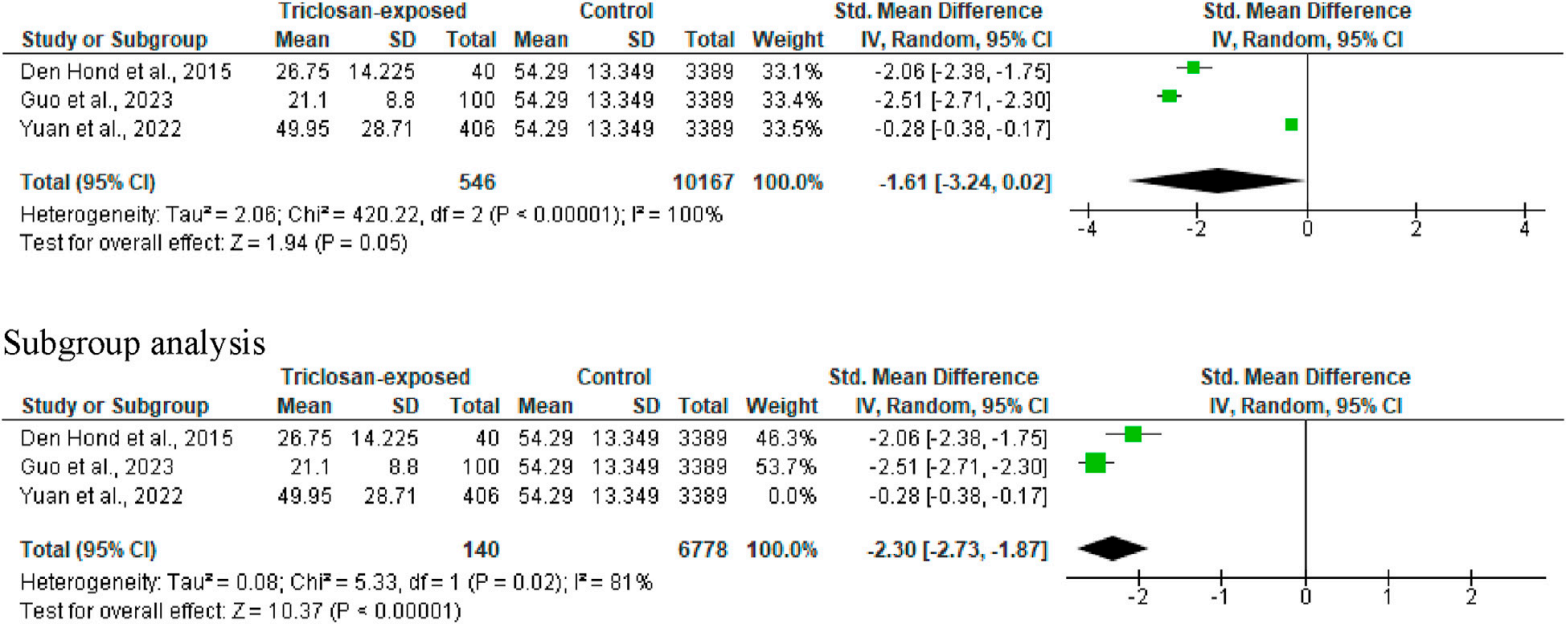
Forest plot showing the effect of triclosan exposure on sperm progressive motility (%).

Three studies were included in the analysis of progressive sperm motility. Triclosan reduced progressive sperm motility (SMD -1.61 [95% CI: 3.24, 0.02] *p* = 0.05). In addition, the inter-study heterogeneity was significant (I^2^ = 100%; X^2^ *p* < 0.00001). Surprisingly, after a subgroup analysis, it was observed that triclosan significantly reduced progressive sperm motility (SMD -2.30 [95% CI: 2.73, −1.87] *p* < 0.00001), and the significant inter-study heterogeneity also persisted (I^2^ = 81%; X^2^ *p* = 0.02) (Figure 7).

#### 3.3.6 Normal sperm morphology

**FIGURE 8:**
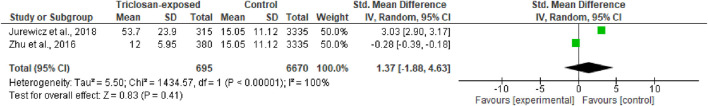
Forest plot showing the effect of triclosan exposure on normal sperm morphology (%).

Two studies were included in the analysis of progressive sperm motility from the eligible studies. Triclosan did not alter sperm morphology (SMD 1.37 [95% CI: 1.88, 4.63] *p* = 0.41). Furthermore, the inter-study heterogeneity was significant (I^2^ = 100%; X^2^ *p* < 0.00001) (Figure 8).

## 4 Discussion

Widespread presence and regular use of triclosan as well as reports linking triclosan exposure to an impairment of the hypothalamic-pituitary-thyroid axis in experimental models and humans [reviewed in 9 and 36] has raised concern that triclosan might adversely affect the male reproductive system. However, while animal and *in vitro* studies have shown that triclosan exposure is associated with altered hormone levels, decreased sperm production, and impaired reproductive organ development, findings from human studies are less clear. Therefore, the present study aimed to collect currently available evidence from a limited number of epidemiologic studies on the association between triclosan exposure and semen quality parameters. We objectively quantified this effect to obtain reliable evidence of potential reproductive triclosan toxicity in the first meta-analysis on this topic.

### 4.1 Main findings

Using a search strategy and criteria for eligibility based on the PECOS framework, and following the PRISMA standards, results of our meta-analysis performed on five articles with data from 1,312 male adult subjects demonstrated that triclosan has the potential to decrease semen quality in men. The most sensitive parameters were sperm concentration and motility, the most commonly used parameters in the evaluation of male factor infertility (Sikka and Hellstrom, 2016). Our results showed that triclosan exposure significantly reduced sperm concentration, even after subgroup and sensitivity analyses. A significant impact of triclosan on semen motility was not confirmed in sensitivity analysis. Triclosan exposure did not significantly alter sperm count and sperm morphology.

Sperm concentration was measured in all five eligible studies, and three of them found a significant decrease in sperm concentration (Den Hond et al., 2015; Jurewicz et al., 2018; Guo et al., 2023). However, inter-study heterogeneity among these studies (I^2^ = 97%) was very high (Figure 4). To find a possible source of the heterogeneity, subgroup and sensitivity analyses were performed. As quality analysis following the GRADE guideline (Table 4) indicated that cohort studies were less eligible than case-control studies, the subgroup analysis was performed by omitting cohort studies (Yuan et al., 2022; Zhu et al., 2016; Jurewicz et al., 2018) in the repeated meta-analysis. The resulting SMD remained significant, however, heterogeneity was still quite high. High heterogeneity may be caused by a small number of eligible studies (together with a limited number of epidemiologic studies on this topic in the literature) and the variability in study population selection, triclosan exposure levels, presented semen quality parameters, confounding factors, statistical methods, and the way how measured parameters were presented. The most likely reason for this very high heterogeneity is the small number of eligible studies included in the meta-analysis. In addition, small sample size, particularly in the case-cohort studies, and differences in study population characteristics, might contribute to the high inter-study heterogeneity. Therefore, further good-quality and comparable studies are required to provide more conclusive data regarding the effects of triclosan exposure on human semen quality.

The conclusions of the other review articles assessing the effect of triclosan exposure on measures related to semen quality vary depending on the included publications. Zamkowska et al. (2018) included three studies (Chen et al., 2013; Den Hond et al., 2015; Zhu et al., 2016) in their narrative review and concluded that triclosan exposure may affect semen quality parameters. This conclusion is based on the results obtained by Zhu et al. (2016) who found an inverse association between triclosan exposure and sperm concentration, total sperm count, number and percentage of sperm with normal morphology among men with the lowest tertile of urinary triclosan levels. The other two included studies found no association between triclosan exposure and semen volume, sperm concentration, and total sperm count (Chen et al., 2013) or sperm concentration, motility, and morphology (Den Hond et al., 2015). Summarizing the available evidence of the association between triclosan exposure and human infertility (Chen et al., 2013; Den Hond et al., 2015; Zhu et al., 2016; Jurewicz et al., 2018; Nassan et al., 2019; Smarr et al., 2018), Daza-Rodríguez et al. (2023) concluded that there is insufficient data to support the hypothesis that triclosan causes infertility in humans although some semen quality parameters might be susceptible to triclosan exposure. A possible explanation for these inconsistencies between studies could be the difference in exposure level and duration considering the observed declines in triclosan concentrations following the triclosan ban in 2016. Furthermore, it is interesting to note that several studies found a non-monotonic dose-response relationship between triclosan levels in urine or seminal plasma and semen quality parameters (Yuan et al., 2022; Zhu et al., 2016; Jurewicz et al., 2018; Guo et al., 2023). For example, an inverse association between triclosan and sperm concentration/sperm count or number/percentage of sperm with normal morphology was observed in the participants with lower triclosan exposure, but stayed almost the same at higher triclosan levels (Zhu et al., 2016). The non-monotonic dose-response relationship is one of the characteristics of endocrine-disrupting chemicals that interfere with hormonal systems acting by different modes of action at different dose levels.

### 4.2 Potential mechanisms underlying triclosan-induced reproductive toxicity

Similar to non-steroidal estrogens such as bisphenol a (BPA) and diethylstilbestrol (DES), the chemical structure of triclosan resembles 17β-estradiol (Wolstenholme et al., 2011; Radwan et al., 2021), leading to the assumption that this compound may act as an endocrine-disrupting agent. Nevertheless, unlike a vast array of papers revisiting the impact of other endocrine disruptors on substandard semen quality through different parts of the endocrine system, the current knowledge of triclosan exposure by and large remains obscure, mostly due to a lack of more comprehensive human studies. To provide additional clarity on the acknowledged controversial effects of triclosan on semen quality in humans, *in vitro* and experimental studies in animal models were explored to evaluate the mechanism of triclosan toxicity on human semen due to the limited data from human studies.

Pivotal *in vitro* studies on triclosan took advantage of carcinoma cell lines (MCF7, T47D, JEG-3) that are excellent models for studying estrogen synthesis and response genes. By up-regulating the expression of pS2, a downstream target gene of estrogen, and down-regulating ERα expression at the mRNA and protein levels, triclosan has been shown to have estrogenic and anti-androgenic properties (Gee et al., 2008; Huang et al., 2014; Yoon and Kwack, 2021). At the same time, triclosan increased the concentrations of endogenous miR-22, miR-206, and miR-193b, hence reducing ERα expression through these microRNAs (Huang et al., 2014). Triclosan reduced the survival of mouse TM3 Leydig cells and blocked the Map3k1/JNK/c-Jun pathway, which is crucial for controlling steroidogenesis in response to different extracellular stimuli (Ha et al., 2018). The induction of miR-6321 expression, which results in the translational repression and degradation of Map3k1, which is necessary for JNK activation and c-Jun phosphorylation, is the molecular mechanism responsible for this regulation. As a result, the orphan nuclear receptor Nur77 as an early response gene expressed in hormonally stimulated steroidogenic cells (Ha et al., 2016), and with crucial roles in the transcription of steroidogenic enzymes and testosterone production will be impaired (Lee et al., 2009). Consequently, SRB1, steroidogenic acute regulatory protein (StAR), and 3β-hydroxysteroid dehydrogenase (3β-HSD) will be suppressed in TM3 cells following triclosan exposure. This phenomenon was additionally accompanied by the downregulation in 5α-reductase, most likely as a compensatory mechanism to compensate for the detrimental effects of triclosan on testicular steroidogenesis (Ha et al., 2018).

**FIGURE 9:**
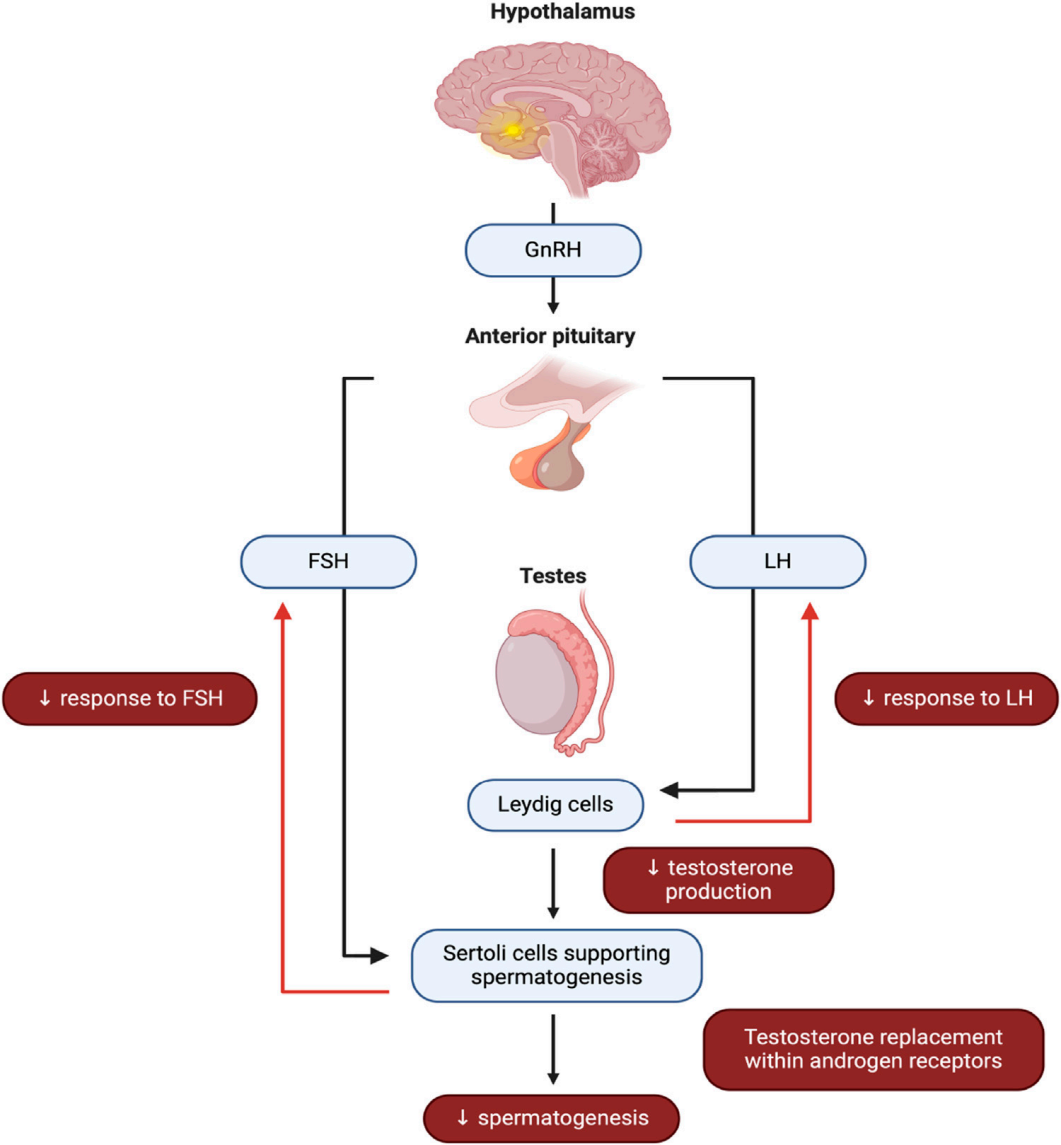
Commonly observed effects of triclosan on the hypothalamic-pituitary-testicular axis.

In animal studies, the ability of triclosan to interfere with the endocrine system has been well documented by a decrease in FSH, LH, and testosterone, indicating a disturbance of the hypothalamic-pituitary-testicular (H-P-T) axis (Figure 9). Moreover, Ha et al. (2018) observed a decline of the luteinizing hormone/choriogonadotropin receptor as well as the androgen receptor in the testicular tissue of rats exposed to triclosan serving as two vital receptors in the activation of the H-P-T loop. As revealed by Kumar et al. (2008), triclosan presents with the ability to cease cAMP synthesis as a consequence of adenylyl cyclase disruption. Since cAMP acts as a crucial second messenger to the LH signal for testosterone synthesis through pregnenolone, it may be speculated that triclosan may decrease the response of the Leydig cells to pituitary action, and hence fail to stimulate the seminiferous tubules to produce spermatozoa (Schlaff and Wierman, 1990). Moreover, triclosan has been shown to possess a high ability to inhibit human CYP19A1 which encodes aromatase. Li et al. (2017) report that triclosan binds to the testosterone binding pocket and the steroid-active sites to competitively block aromatase, which may result in relative infertility. This was discovered through a docking study utilizing human CYP19A1 (Bulun, 2014). However, another possible way that triclosan disrupts the H-P-T axis and ultimately prevents testosterone synthesis is by competitively binding to the receptor, which displaces testosterone from its native hormone receptor (Wang and Tian, 2015). Furthermore, it has been documented that rats exposed to triclosan exhibit transcriptional downregulation of key enzymes in testosterone biosynthesis, including 3β-HSD, 17β-hydroxysteroid dehydrogenase (17β-HSD), cytochrome P450 side chain cleavage (P450SCC), cytochrome P450c17, and StAR (Kumar et al., 2009). On the other hand, increased levels of Sult1e1, Ugt1a1, and 5α-reductases involved in steroid hormones metabolism observed by Feng et al. (2016) suggests an additional burden to the testosterone homeostasis due to an increased loss of the male regulator. Finally, triclosan metabolites may exhibit the most potent endocrine-disrupting effect on thyroid hormones (Crofton et al., 2007).

The upregulation of mRNA expression for complement 3, an estrogen-sensitive gene (Jung et al., 2012; Marques et al., 2022), calbindin-D9k (CaBP-9k), a biomarker for the detection of endocrine disruptors in GH3 cells (Marques et al., 2022; Louis et al., 2013), and estrogen production in the H295R steroidogenesis assay (Farmer et al., 2018) have also been linked to the endocrine disrupting activities of triclosan. Hence, it is likely that the observed reduction in semen quality following triclosan exposure was mediated, at least in part, by endocrine disruption, possibly *via* increased estrogen production and reduction of testosterone levels (Farmer et al., 2018).

All molecular effects of triclosan observed at the level of the endocrine system were furthermore accompanied by histopathological changes in the testicular and epididymal tissue. The most common alterations included decreased height of the germinal epithelium and disorganized and detached germ cells in the seminiferous tubules (Lan et al., 2015; Raj and Singh, 2023), vacuolated and exfoliated epithelial cells (Mahmoud and Solaiman, 2014). Furthermore, sperm tails were absent from the lumen of the seminiferous tubules (Lan et al., 2015). Ultra-structurally, triclosan treatment led to deformed nuclei and aggregated chromatin, significant intracellular vacuolization, swollen mitochondria, and dilated rough endoplasmic reticulum were observed in the germinal epithelium (Ha et al., 2018). Testicular degeneration, accompanied by severe alterations in testosterone biosynthesis may therefore at least partially explain the compromised capacity of the male reproductive system to effectively produce male gametes and may account for the significant reduction of sperm concentration in the analyzed studies.

Subsequent impairment of the sperm quality and fertilizing ability as a result of triclosan toxicity may be linked to different mechanisms out of which mitochondrial dysfunction, reactive oxygen species (ROS) production, and associated oxidative balance, apoptosis, and sperm size have been proposed by previous studies, particularly in rodents (Raj et al., 2021; Chigrinets et al., 2020; Montagnini et al., 2021) and aquatic species (Rolton et al., 2022).

**FIGURE 10:**
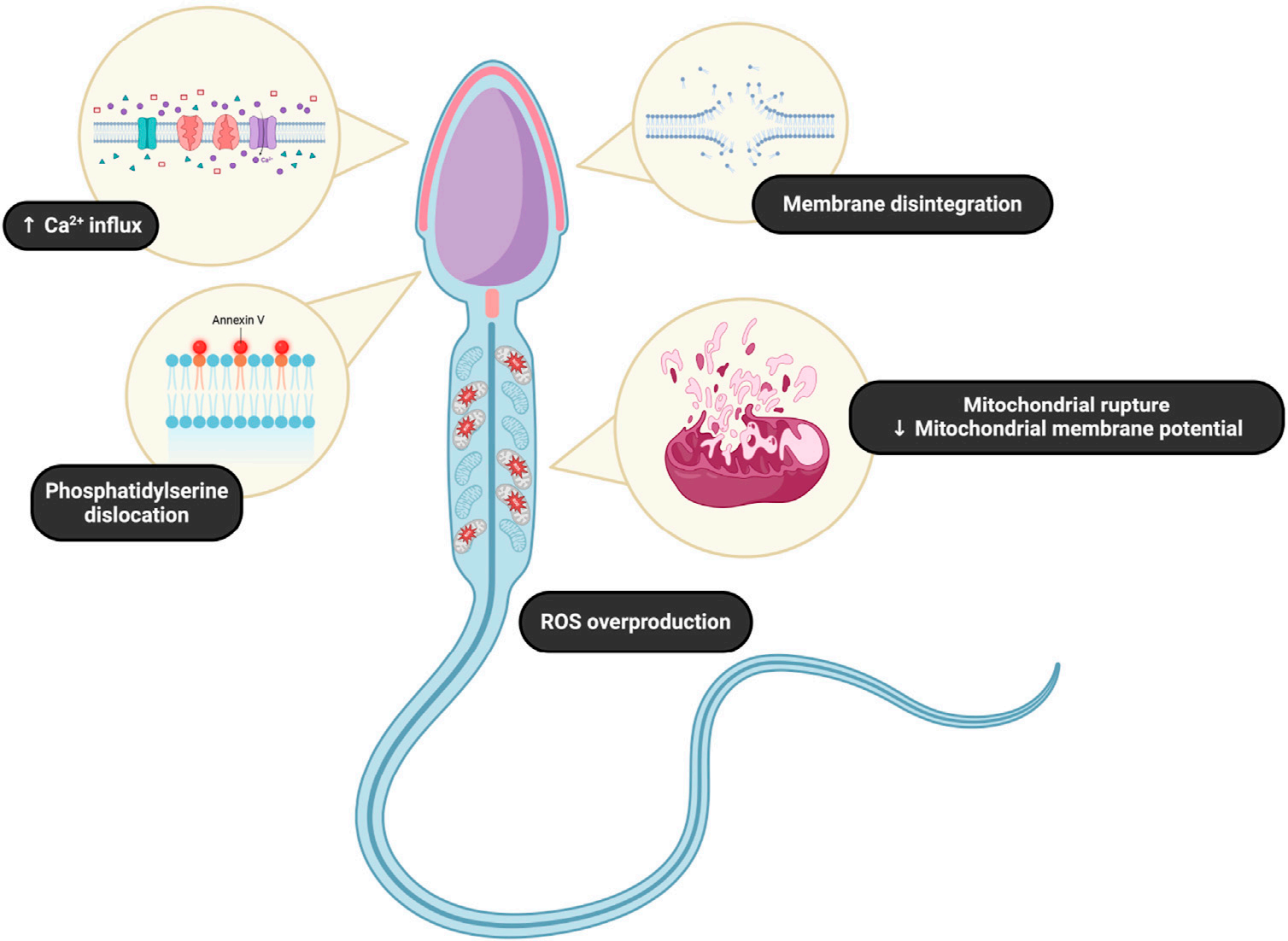
Commonly observed effects of triclosan on spermatozoa.

Previous studies have unraveled the membrane-destabilizing effects of triclosan, leading to compromised structural integrity of spermatozoa (Binelli et al., 2011; Villalaín et al., 2001). High expression of proteins involved in the stress response of spermatozoa alongside annexin overexpression has been reported in spermatozoa of animal subjects exposed to triclosan (Rolton et al., 2022; Riva et al., 2012). Since annexins are calcium-binding proteins that regulate its influx and efflux from and to the cytoplasm, the disruption of intracellular calcium homeostasis could lead to cellular swelling (Rolton et al., 2022), the loss of mitochondrial membrane potential and subsequent mitochondrial dysfunction, ROS leakage, and apoptosis (Riva et al., 2012; Wang et al., 2020) (Figure 10).

Mitochondria as the center of cellular respiration and energy synthesis for the sperm movement ought to be the prime structure susceptible to triclosan toxicity since they are also involved in calcium signaling and the intrinsic apoptotic machinery (Amaral et al., 2013). At the same time, the organelles are principal ROS producers as a by-product of respiration. While physiologically appropriate ROS levels act as signaling molecules involved in critical cellular functions that promote sperm capacitation, hyperactivation, acrosome reaction, and gamete fusion, their overgeneration leads to oxidative damage to structures paramount to sperm survival (Benko et al., 2022). This phenomenon is furthermore complicated by the fact that spermatozoa are characterized by a high surface: area ratio, their antioxidant activity is limited due to the small amount of cytoplasm, and their membranes are primarily composed of polyunsaturated fatty acids. As such, excessive ROS levels observed in previous reports suggest their incomplete scavenging or the antioxidant mechanisms may have been overwhelmed. Accordingly, high levels of lipid peroxidation products have been observed in the reproductive cells obtained from subjects exposed to triclosan (Riva et al., 2012; Freitas et al., 2019; Webb et al., 2020). Hence, the susceptibility of sperm cells to triclosan attack may be due to its high content of polyunsaturated fatty acids that are easily oxidized by ROS (Besong and Akhigbe, 2024; Adeogun et al., 2024; Ashonibare et al., 2024; Akhigbe et al., 2024a). On this account, it seems plausible that mitochondrial dysfunction may be the prime reason for the low sperm motility unraveled in the studies included in this meta-analysis. To confirm this theory, more epidemiological studies with a special focus on the effects of triclosan on the seminal oxidative balance and mitochondrial function are necessary. Although triclosan differs from other environmental toxicants in chemical structure, it exerts similar effects on sperm quality by eliciting oxidative stress (Besong et al., 2024; Akhigbe et al., 2024b; Akhigbe et al., 2024c; Hamed MA. et al., 2023). Triclosan is lipophilic and thus accumulates in fatty tissues. This property could affect concentrations in various body fluids, including semen, which may reflect systemic exposure (Pan et al., 2024).

### 4.3 SWOT analysis

**FIGURE 11:**
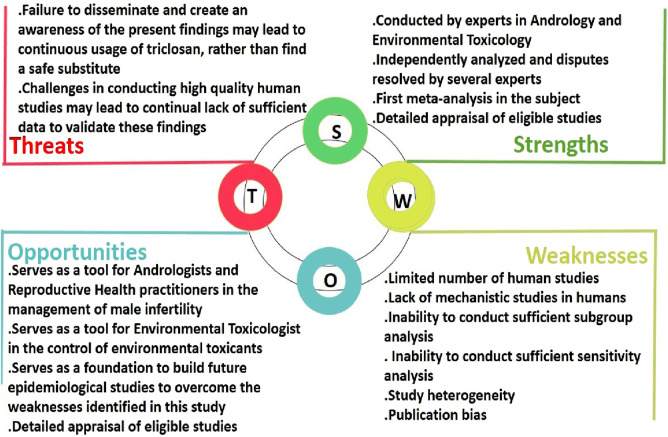
The strengths, weaknesses, opportunities, and threats (SWOT) analysis of the present study on the impact of triclosan exposure on human semen quality.

The strengths, weaknesses, opportunities, and threats of the present study are highlighted in Figure 11. This summarizes the advantages and limitations of the present meta-analysis. Also, opportunities that could be harnessed from this study in the medical and scientific community are highlighted. Although the paucity of available articles exploring the impact of triclosan on semen quality limited the subgroup and sensitivity analyses, findings of the subgroup and sensitivity analyses conducted revealed that the sources of heterogeneity are promarily the sample size and study design. The sample size influenced the weight and the magnitude of the effect being measured, resulting in a change in the impact of triclosan on total sperm motility after sensitivity analysis. Also, the study design influenced the outcome observed following subgroup analysis (that included only case-controlled studies) of the effect of triclosan on progressive sperm motility. However, the study presents a robustness of results under different statistical assumptions. The subgroup and meta-analysis revealed the sources of heterogeneity and strengthened our findings.

## 5 Conclusions and future perspectives

This study is the first meta-analysis to evaluate the impact of triclosan on semen quality in men. Although there are currently few studies on triclosan’s reproductive toxicity in men that could be included in the meta-analysis, our results suggest that triclosan may have a significant impact on semen quality by reducing sperm concentration and motility. However, we are unable to offer more definitive information about the impact of triclosan exposure on human semen quality, due to the significant heterogeneity found among the included research.

Epidemiologic studies on semen quality effects of triclosan exposure are sparse and include only a limited number of semen quality and reproductive hormone parameters. Furthermore, it is challenging to link changes in semen quality exclusively to triclosan due to small sample sizes, differences in study population characteristics, exposure levels, and duration, study design, simultaneous exposure to other chemicals, and lifestyle factors. Therefore, large-scale, well-designed studies in various geographic regions are needed to elucidate the possible connections between triclosan exposure and male infertility.

In the absence of proper studies on biochemical and molecular mechanisms underlying triclosan reproductive toxicity in humans, this information was obtained from the experimental studies. *In vitro* and animal studies indicated that triclosan-induced reduction in semen quality is mediated by the activation of oxidative stress signaling pathways. Since the mechanistic insights are largely based on animal and *in-vitro* studies, it is worth noting that extrapolating these findings to humans should be done with caution and further mechanistic studies in humans are recommended. Future epidemiologic studies should also focus on the mechanism of action through which triclosan could promote adverse reproductive effects in men. There is also a need for longitudinal studies and more precise measurements of triclosan exposure to determine the adverse effect of triclosan on semen quality.

## Data Availability

The original contributions presented in the study are included in the article/supplementary material, further inquiries can be directed to the corresponding authors.
