# Supplementary material for: A systematic review and meta-analysis of the impact of triclosan exposure on human semen quality
Source: Front Toxicol. 2024 Oct 17;6:1469340. doi: 10.3389/ftox.2024.1469340 (PMC11525012; doi:10.3389/ftox.2024.1469340)
Supplement: Supplementary file 1 [file DataSheet1.doc]

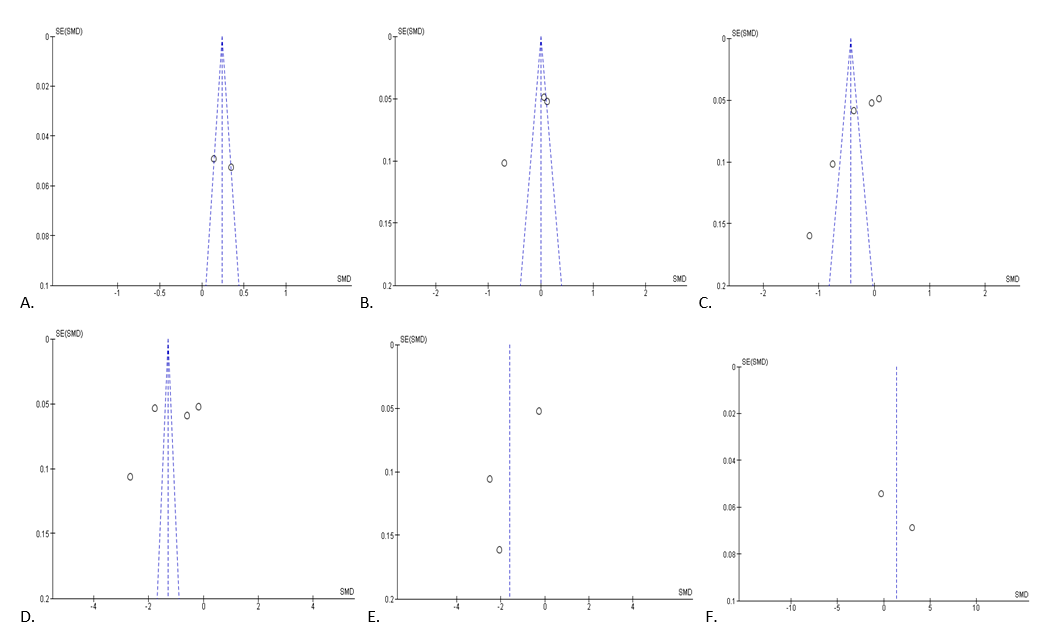


**Supplementary Figure 1**: Funnel plot showing the publication bias for semen volume (A), sperm count (B), sperm concentration (C), total motility (D), progressive motility (E), and normal sperm morphology (F) related to the effects of triclosan exposure. The estimated effect size (Standard Mean Difference, SMD) is plotted on the x-axis and its standard error on the y-axis.
